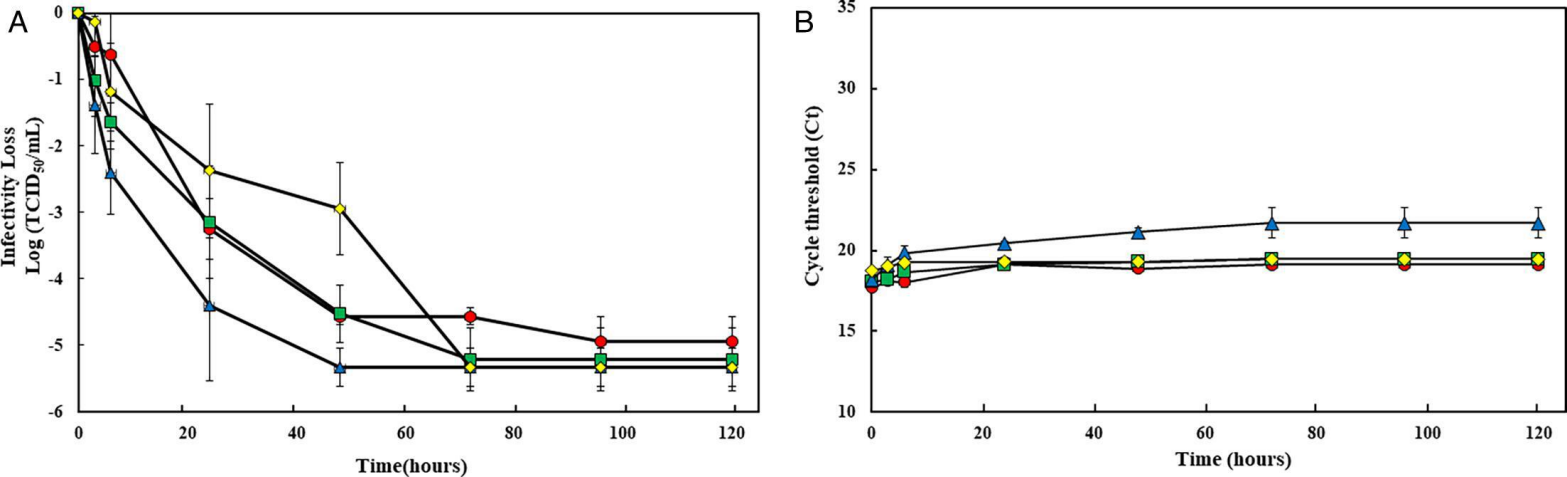# Articles of Significant Interest in This Issue

**DOI:** 10.1128/aem.00766-25

**Published:** 2025-04-23

## ANTIMICROBIAL PEPTIDES, FROM DISCOVERY TO MARKET

This minireview by Qi Zhang (e02115-24) summarizes recent developments in the search for antimicrobial peptides that can realistically provide therapeutic alternatives to antibiotics. Important read for those interested in solutions to the antibiotic resistance crisis.


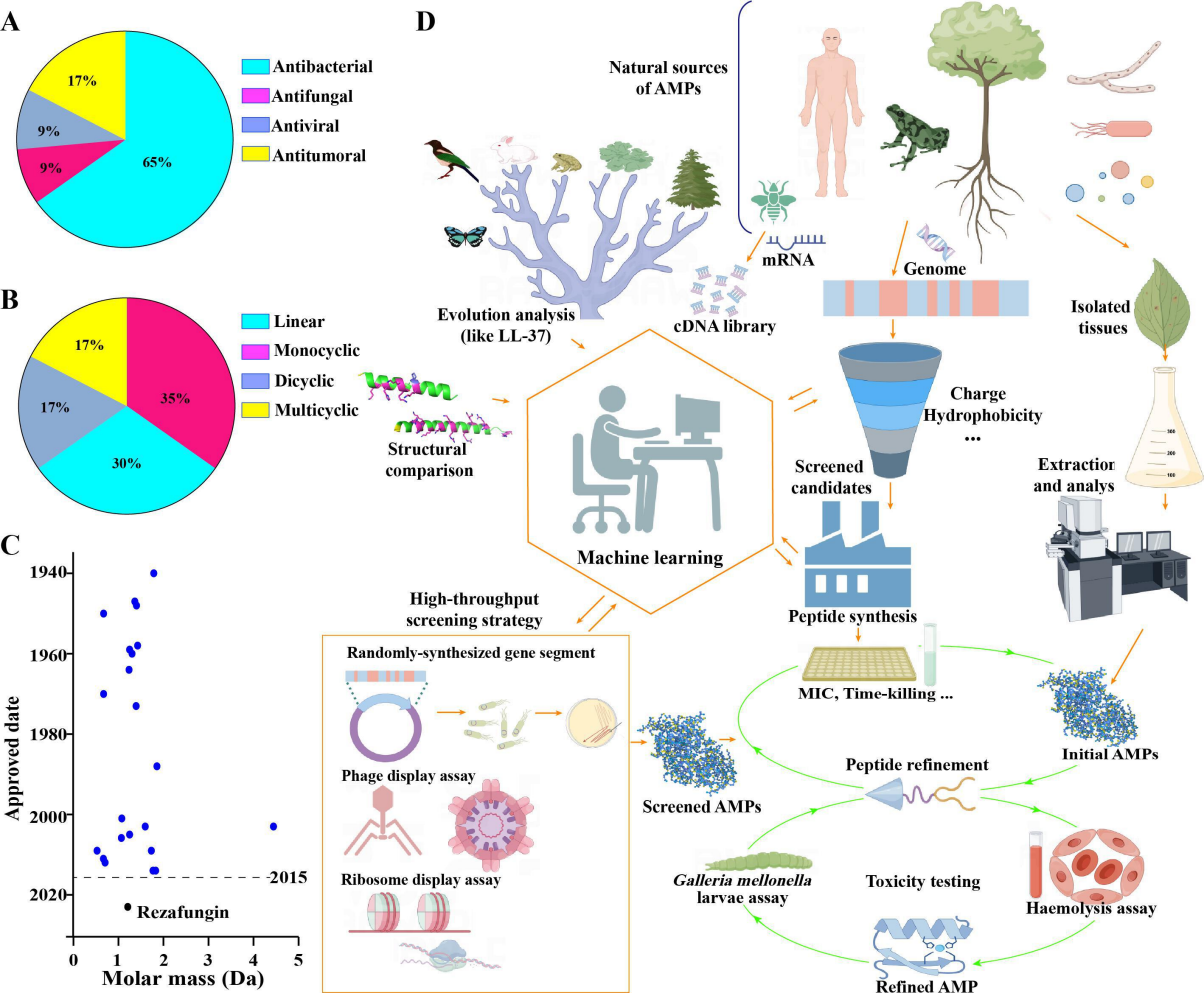


## A HYDROGEN ISOTOPIC VIEW OF ARCHAEAL LIPIDS

This study by Harris et al. (e01983-24) fills a critical gap in our understanding of hydrogen isotope fractionation in archaeal lipids under various extreme conditions and underscores the method’s utility to survey archaea in habitats where physicochemical extremes prevent other approaches.


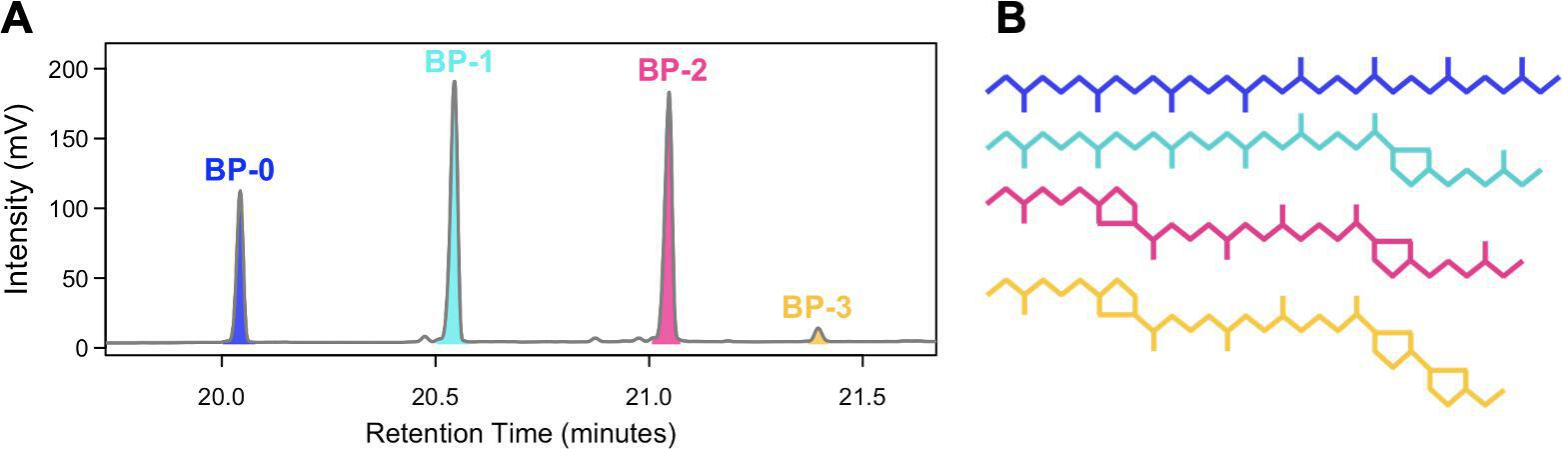


## AN EVOLUTIONARY TALE OF INSECT ENDOSYMBIONTS AND YEAST COMPETITORS

Symbionts in sap-feeding insects support the host’s nutritional needs. A comparative genomic study by Han et al. (e01738-24) of treehopper symbionts dissects the evolutionary steps that have led to the replacement of obligate partners by coexisting yeast-like symbionts.


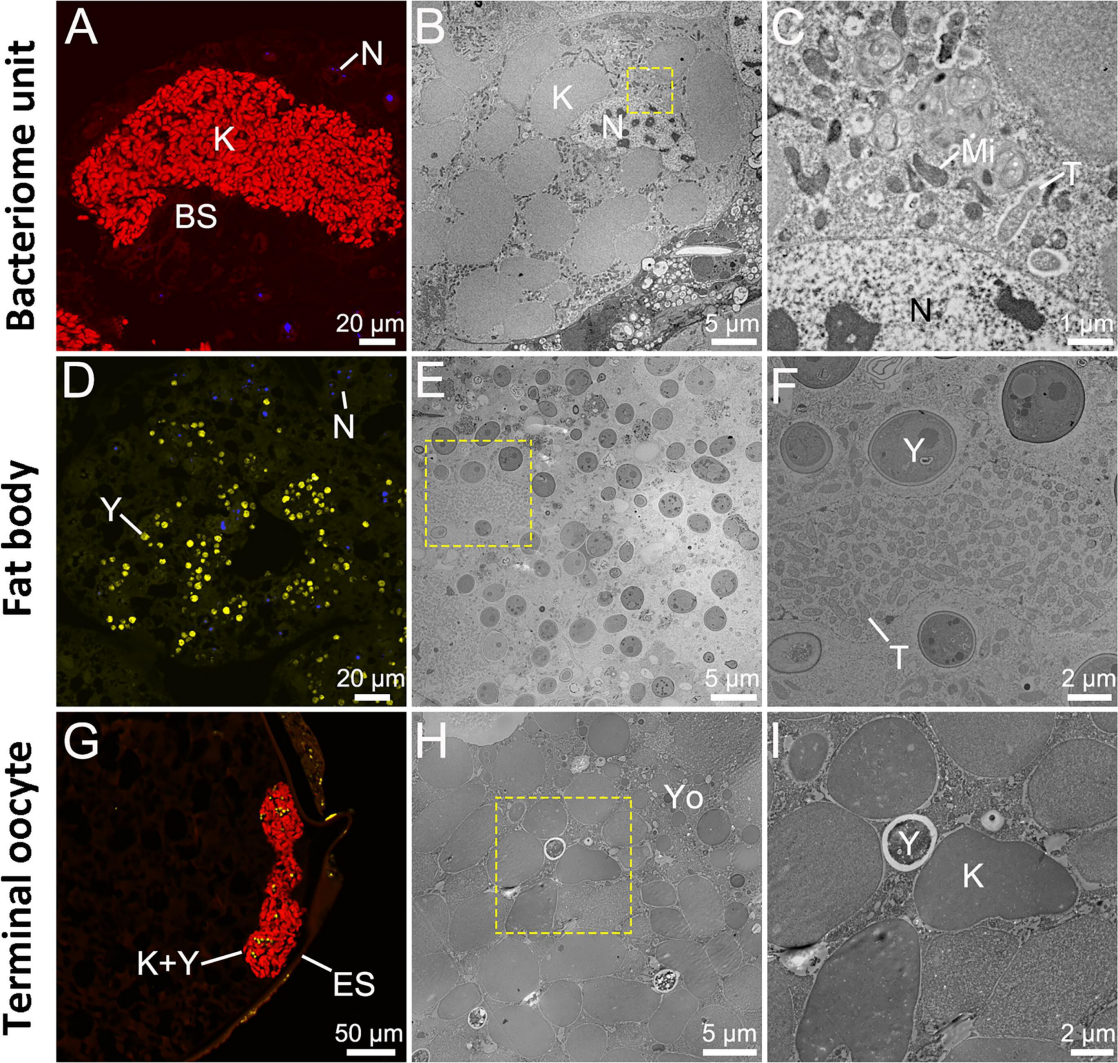


## IRON OXIDATION GOES MAGNETIC

Iron-reducing and -oxidizing bacteria cooperate to cycle this important element in nature. Keffer et al. (e01865-24) show that iron oxidizers also have specialized electron transfer chains to oxidize magnetite, a reduced product of iron reducers of mixed iron valence.


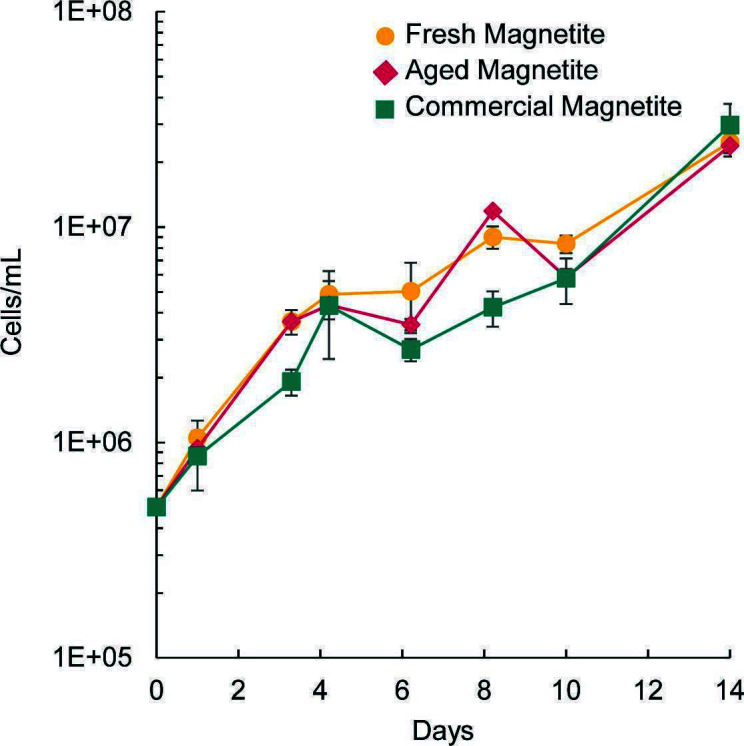


## A PROTIST ALLY FOR THE BIOCONTROL OF PLANT PATHOGENS

Predatory protists show promise as biocontrol agents in crop production systems. Amacker et al. (e00240-25) identify protist species and amendment conditions for consistent and predictable improvements in plant performance.


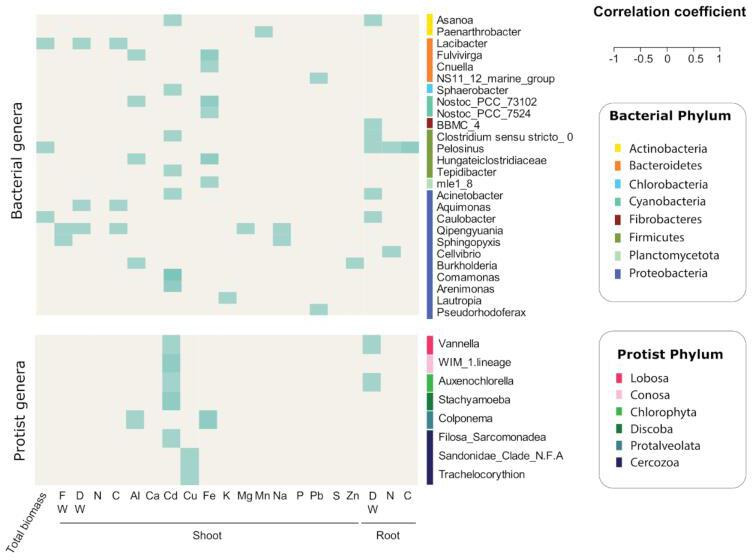


## A CYANOBACTERIAL SURVIVAL GUIDE TO HIGH CO_2_ STRESS

Cyanobacteria are promising chassis for CO_2_ conversion applications, yet they cannot tolerate high concentrations of the gas. Mu et al. (e00115-25) describe regulatory networks driving the cyanobacterial response to high-CO_2_ stress. These insights help us to understand the ecophysiology of these bacteria and inform strategies for the development of robust industrial strains.


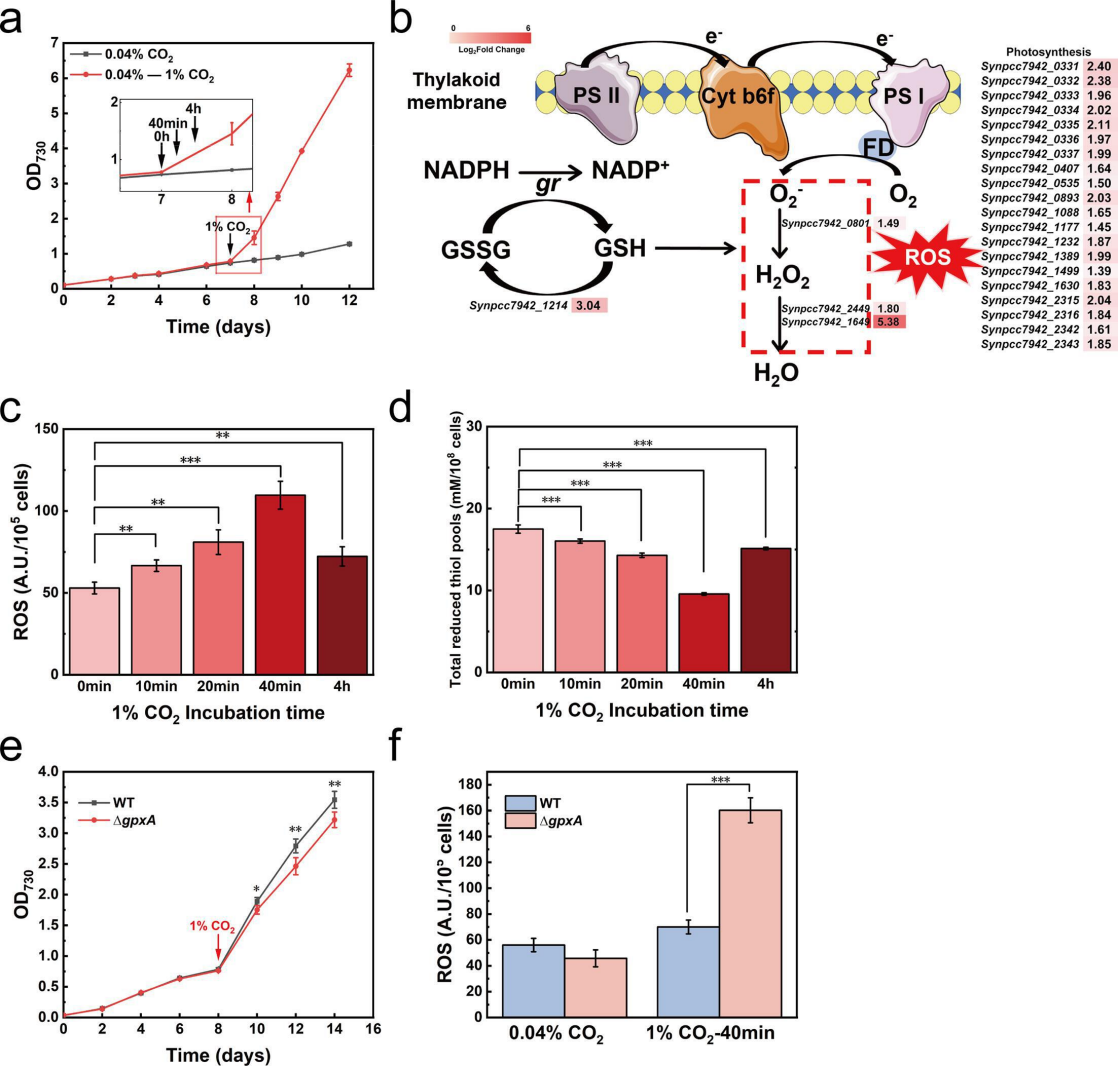


## RHIZOBACTERIA: SELF-PRESERVATION WITH POSITIVE PLANT OUTCOMES

Rhizobacteria produce an indole-based phytohormone to promote plant growth. Ganusova et al. (e02384-24) show that by making the hormone, rhizobacteria detoxify indole. A selfish act with notable benefits to plants and humans.


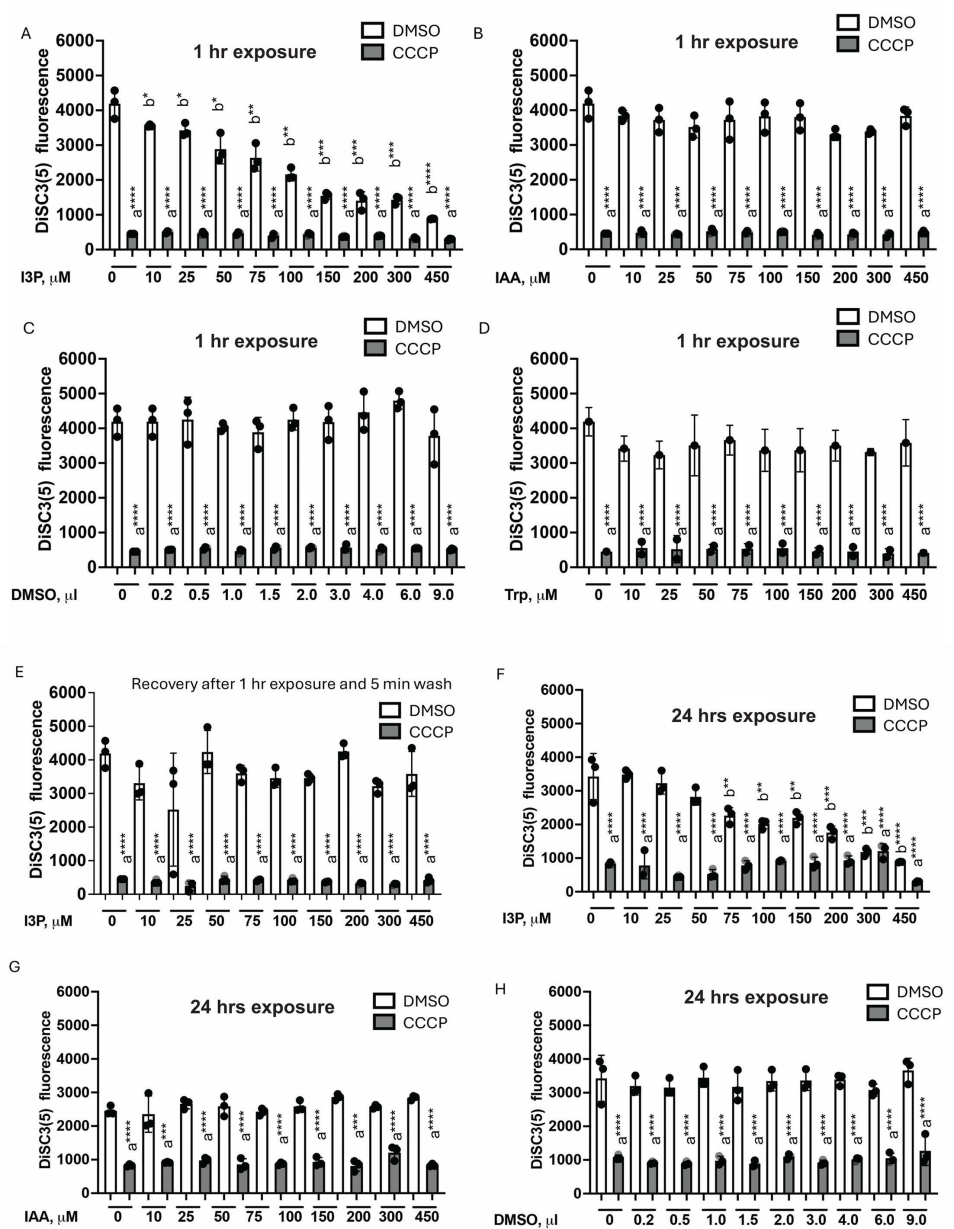


## SARS-CoV-2 SURROGATE CORONAVIRUSES FOR ENVIRONMENTAL SURVEYING

Park et al. (e01688-24) describe coronaviruses that can be used as surrogates for SARS-CoV-2 seasonal surveying. Caution is needed when generalizing the results, however, due to the variable stability of the most promising strains under certain environmental conditions.